# Fibronectin Connecting Cell Sheet Based on Click Chemistry for Wound Repair

**DOI:** 10.1002/advs.202306746

**Published:** 2024-01-02

**Authors:** Wei Xu, Meng He, Qinghua Lu

**Affiliations:** ^1^ School of Chemistry and Chemical Engineering Frontiers Science Center for Transformative Molecules the State Key Laboratory of Metal Matrix Composites Shanghai Jiao Tong University Shanghai 200240 China

**Keywords:** azide group, cell sheet, click chemistry, fibronectin, NIR‐responsive substrates, wound repair

## Abstract

As a living repair material, cell sheet exhibits significant potential in wound repair. Nonetheless, wound healing is a complicated and protracted process that necessitates specific repair functions at each stage, including hemostasis and antibacterial activity. In this work, on the basis of harvesting the cell sheet via a photothermal response strategy, a fibronectin attached cell sheet (FACS) is prepared to enhance its wound repair capability. For this purpose, the azide group (N_3_) is initially tagged onto the cell surface through metabolic glycoengineering of unnatural sugars, and then the conjugate (DBCO‐fibronectin) comprises of the dibenzocyclooctyne (DBCO) and fibronectin with multiple wound repair functions is linked to N_3_ using click chemistry. Biological evaluations following this demonstrates that the FACS preparation exhibits excellent biocompatibility, and the fibronectin modification enhances the capacity for cell proliferation and migration. Moreover, in vivo wound healing experiment confirms the reparative efficacy of FACS. It not only has a wound closure rate 1.46 times that of a conventional cell sheet but also reduces inflammatory cell infiltration, promotes hair follicle and blood vessel regeneration, and encourages collagen deposition. This strategy holds enormous clinical potential and paves the way for advanced functional modifications of cell sheets.

## Graphical abstract

This paper proposes a drug‐functionalization strategy for cell sheet based on click chemistry, where the prepared fibronectin attached cell sheet (FACS) combines the wound repair capabilities of cell sheet and fibronectin, exhibiting a healing rate 1.46 times that of conventional cell sheet and improved tissue regeneration. This strategy holds immense clinical potential.

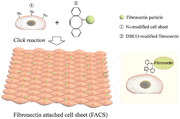

## 1 Introduction

As the largest organ, the skin covers the surface of the human body and plays a crucial role in isolating infringement, metabolic exchange, and temperature regulation.^[^ ^1^, ^2^ ^]^ However, due to its direct contact with the external environment, it is highly vulnerable to damage.^[^ ^3^ ^]^ Although most skin injuries can recover over time, it is primarily infants who have the ability to fully regenerate skin functions, while adults frequently experience issues such as scarring and loss of sweat gland functions.^[^ ^4^ ^]^ Wound healing is an extended and continuous process, encompassing four stages: hemostasis, inflammation, proliferation, and remodeling.^[^ ^5^ ^]^ Factors that disrupt any of these stages may result in abnormal wound healing, characterized by excessive inflammation or infection.^[^ ^6^ ^]^ Thus, additional protection against bacterial invasion or physical damage during wound healing is clinically necessary.^[^ ^7^ ^]^ Among protective measures, wound dressings cover the wound and promote healing, mainly including materials such as gauze, hydrogels, and foam.^[^ ^8^, ^9^ ^]^ Despite the extensive range of wound dressings available, many healing requirements are still not adequately met by current options.^[^ ^10^ ^]^

With high cell density and complete extracellular matrix (ECM), cell sheet engineering has the advantages of good curative effect and high accuracy in wound healing.^[^ ^11^, ^12^ ^]^ Furthermore, autologous cell sheets circumvent complications like immune rejection and postoperative degradation. As such, cell sheets have been applied in various fields, including periodontal regeneration, burn treatment, and cardiac repair.^[^ ^11^, ^13^, ^14^ ^]^ However, cell sheets may not be as therapeutically effective as certain drugs or biochemical agents at specific stages of wound healing. For example, fibronectin not only promotes platelet aggregation but also serves as a pathway for cell migration and a framework for tissue contraction during the proliferation phase.^[^ ^15^, ^16^ ^]^ In addition, agents such as the anticoagulant heparin and antibacterial antibiotics play crucial roles in the healing process.^[^ ^17^, ^18^ ^]^ Therefore, integrating pharmaceuticals with cell sheets may be the most effective method to optimize wound repair efficacy.

**Figure 1:**
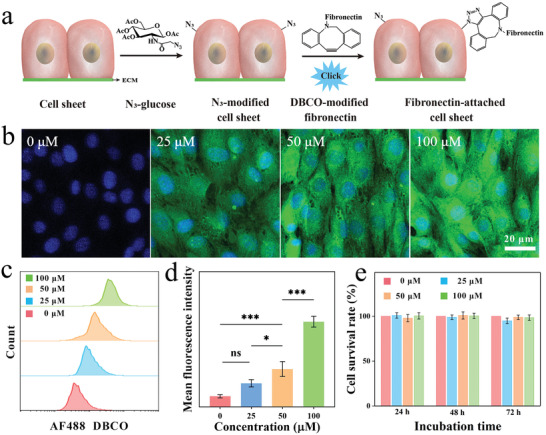
a) Scheme of attaching fibronectin onto cell sheet surface via click chemistry. b) Fluorescence images of N_3_‐glucose tagged onto the cell surfaces with gradient concentrations (0, 25, 50, and 100 µm) stained by AF488 DBCO and Hoechst 33342. c) Corresponding flow cytometric analysis and (d) mean fluorescence intensity. e) Cell survival rates at different time points of N_3_‐glucose tagging were measured via CCK‐8. Statistical significance was calculated by a two‐tailed Student's *t*‐test, ^*^ *p* < 0.05, ^***^ *p* < 0.001, ns indicates no significant difference (Mean ± SD, *n* = 4).

The metabolic glycan tagging technique has emerged as a simple and powerful tool for tagging cell surfaces with chemical tags, whereby chemically modified nonnatural sugars enter cells undergo a series of sugar metabolism processes, and are ultimately expressed on the cell membranes in the form of glycoproteins.^[^ ^19^ ^]^ These chemical tags (such as N_3_, alkynes, etc.) can be linked to targeted drug molecules by click chemistry, exemplified by the reaction between N_3_ and DBCO.^[^ ^20^ ^]^ As shown in **Figure** **1**a, this work proposed a drug functionalization strategy for cell sheets by attaching fibronectin onto the cell sheet surface to improve its wound healing capability. In detail, a dynamic wrinkling biointerface (Wrinkle‐Col) was prepared as previously reported, and the cell sheet was harvested under near‐infrared light (NIR) irradiation.^[^ ^21^ ^]^ The N_3_ group was then tagged on the cell sheet surface by metabolic glycan tagging technique and subsequently linked to DBCO‐modified fibronectin via click chemistry to prepare the fibronectin attached cell sheet (FACS). Cell biological studies revealed that fibronectin attachment had no potential toxicity to cells and significantly promoted cell migration and proliferation. In addition, the enhanced wound repair efficacy of FACS was demonstrated by in vivo wound healing experiment. In summary, this drug‐assisted strategy of cell sheet represents a new frontier in cell sheet engineering, potentially advancing its applications in regenerative medicine.

## 2 Results

### 2.1 N_3_ Tagging and Characterization of Cell Surfaces

Cell‐surface tagging is a technique that introduces unique chemical functional groups (CFGs) onto the cell surface to serve as surrogates for glycoprotein receptors. These CFGs can mimic some functions of glycoprotein receptors.^[^ ^22^ ^]^ Among these, the metabolic glycan tagging strategy using unnatural sugars is a simple and powerful technique for cell surface tagging, enabling the easy introduction of CFGs into glycoproteins at desired densities.^[^ ^19^ ^]^ Generally used unnatural sugars include chemically modified azidomannosamine, azidoglucosamine, and azidogalactosamine.^[^ ^19^ ^]^ This work employed gradient concentrations (0, 25, 50, and 100 µm) of azidoglucosamine (N_3_‐glucose) to tag cells, which was then linked to DBCO‐modified drug molecules by click chemistry. The tagging efficiency of the N_3_ group was detected by DBCO‐modified fluorescent dye (AF488 DBCO). As shown in Figure 1b, when the tagging concentration was 0 µm, the cell sheet only exhibited blue fluorescence of the nucleus. With increasing tagging concentration, the cell sheet displayed clear and bright green fluorescence. Further flow cytometry analysis quantitatively measured the changes in fluorescence intensity with the concentration of N_3_‐glucose and calculated the mean ± standard error after data analysis using GraphPad Prism 8.0 software (Figure 1c,d). The increase in mean fluorescence intensity was linearly positively correlated with the corresponding N_3_‐glucose concentration, which fully demonstrated the high tagging efficiency of the N_3_ group within the given concentration range.

To evaluate the impact of N_3_ tagging on cell viability, the tagged cells were characterized using CCK‐8 kit and a microplate reader (Figure 1e). At the same incubation time, the cell survival rates of different tagging concentrations (0, 25, 50, and 100 µm) were all close to 100%, suggesting that N_3_‐glucose addition exerted minimal to no impact on cell viability and that N_3_ tagging exhibited excellent biocompatibility.

### 2.2 The Synthesis of DBCO‐Fibronectin

**Figure 2:**
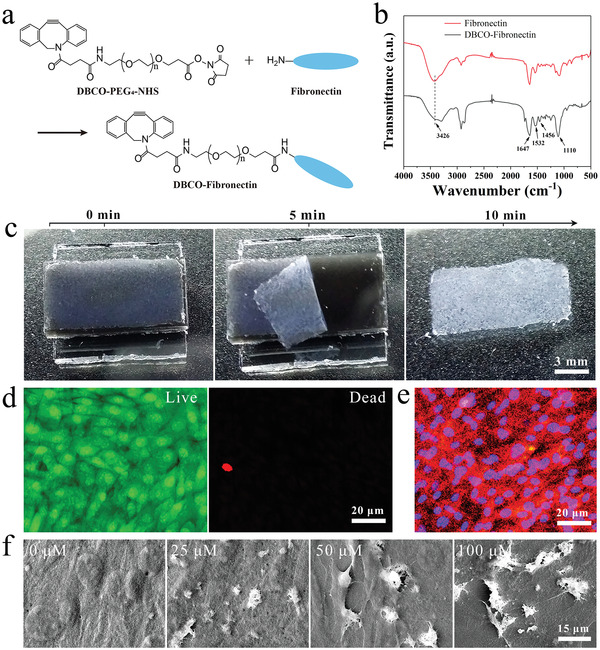
a) The synthesis route of DBCO‐Fibronectin. b) The FTIR spectra of the fibronectin and DBCO‐Fibronectin. c) Optical images of the harvesting process of the FACS. NIR at 3.5 W cm^−2^. d) Fluorescence images of cell viability of the harvested FACS stained by AO/EB. e) Immunofluorescence images of the FACS stained by N‐cadherin (red) and DAPI (blue). f) SEM images of surface topography of the FACS.

To attach the required fibronectin onto the cell surface, diphenylcyclooctyne‐tetraethylene glycol‐active ester (DBCO‐PEG_4_‐NHS ester) was used as a medium, one end of which attaches to the N_3_ group on the cell surface and the other end links to fibronectin. As a derivative of DBCO, the hydrophilic PEG long chain not only increases its solubility in aqueous solutions, but it can also reduce steric hindrance during the reaction. In addition, the terminal NHS ester can form stable covalent bonds with primary amines under neutral or weakly base conditions, such as forming amide bonds with the side amino groups in fibronectin (**Figure** **2**a). Figure 2b shows the Fourier transform infrared (FTIR) spectra of fibronectin and DBCO‐Fibronectin. The peaks at 1647, 1532, and 1456 cm^−1^ are the characteristic peaks of amide bonds (O═C─NH─), attributed to the stretching vibration of C═O, the deformation vibration of N─H and the stretching vibration of C─N, respectively. The intensities of these peaks in the spectrum of DBCO‐Fibronectin were notably higher than those of fibronectin, indicating the formation of new amide bonds. Moreover, the DBCO‐Fibronectin spectrum exhibited the stretching vibration peak of C─O from the PEG fragment at 1110 cm^−1^, along with the weakened peak intensity at 3426 cm^−1^, resulting from the partial conversion of primary amines to secondary amines. To obtain more characterization data, the DBCO‐Fibronectin was also determined by using ^1^H NMR using DMSO‐d_6_ as the solvent. As shown in Figure S1 (Supporting Information), the ^1^H NMR spectrum of DBCO‐Fibronectin revealed the characteristic peak of DBCO at 7–8 ppm, as well as the characteristic peaks of fibronectin at ≈1 and 2.5 ppm. Therefore, the FTIR spectra and ^1^H NMR spectra jointly testified to the successful synthesis of DBCO‐Fibronectin.

### 2.3 The Attachment of the Fibronectin to Cell Surfaces

Click chemistry refers to the simple and efficient synthesis of diverse functional molecules through the splicing of small molecular units under mild conditions, characterized by rapid and selective reactions between functional groups.^[^ ^23^ ^]^ Currently, a series of click chemistry reactions have been developed to ensure non‐interference with the normal biochemical reactions of organisms in bioorthogonal reactions. For instance, reactions between the N_3_ group and cyclooctyne as well as its derivatives proceed without the need for an exogenous catalyst and are non‐cytotoxic.^[^ ^24^, ^25^ ^]^ Therefore, this work utilized the rapid reaction between N_3_ and DBCO to attach fibronectin onto the cell surfaces for preparing FACS. As shown in Figure S2 (Supporting Information), after incubating cells tagged with N_3_ on the surface of Wrinkle‐Col substrates with DBCO‐Fibronectin (0, 25, 50, and 100 µm) for 30 min, the fibronectin particles on the cell surfaces could be clearly seen. As the DBCO‐Fibronectin concentration increased, the attached particles became denser. These particles were firmly bound to the cells and did not fall off when rinsed with PBS. Conversely, the fibronectin as a control, which was not modified by DBCO, fell off easily after a 30‐min incubation with cells when rinsed with PBS. We also attempted to incubate cells not tagged with N_3_ with DBCO‐Fibronectin (100 µm) for 30 min. As a result, the fibronectin fell off easily upon rinsing with PBS (Figure S3, Supporting Information). Therefore, through the reasonable setup of the control group, we were able to demonstrate that N_3_ and DBCO were key for the successful attachment of fibronectin onto the cell surfaces and that the click chemistry reaction between N_3_ and DBCO did occur during this process.

Furthermore, the CCK‐8 kit was used to assess the impact of DBCO‐Fibronectin on cell survival rate (Figure S4, Supporting Information). At various concentrations, the cell survival rates remained relatively stable, showing only a slight decrease after 72 h of incubation. These results unequivocally demonstrate that fibronectin and its attachment process via click chemistry are biocompatible and offer a reference for on‐demand drug delivery using cell sheets in regenerative medicine.

### 2.4 Harvesting and Characterization of FACS

To fully harness the combined benefits of fibronectin and cell sheet, FACS was prepared using the Wrinkle‐Col substrate and photothermal response strategy.^[^ ^21^ ^]^ The cells were first cultured on the Wrinkle‐Col substrate to more than 90% density, then incubated with DBCO‐Fibronectin (100 µm) for 30 min, and the FACS could be harvested under NIR irradiation. As shown in Figure 2c, the harvesting process for the FACS took 10 min. Notably, the FACS exhibited higher roughness in appearance than that of ordinary cell sheets due to the bound fibronectin. Biological evaluations confirmed that >90% of the cells remained highly viability, and the intercellular connections in the FACS were intact (Figure 2d,e). In summary, the NIR‐stimulated harvesting process was biocompatible and preserved the functionality and integrity of the FACS.

To characterize the binding of fibronectin onto the cell surfaces of the FACS after harvesting from the Wrinkle‐Col substrate, the surface topographies were observed with a scanning electron microscope (SEM) (Figure 2f). The white particles of fibronectin were clearly visible and firmly attached to the cell surfaces of the FACS. As the DBCO‐Fibronectin concentration increased, these particles on the cell surfaces became denser, which was consistent with the observations made prior to harvesting the FACS. Thus, fibronectin was effectively bound to the FACS cell surfaces and maintained a strong attachment post‐harvest.

### 2.5 Scratch‐Regeneration Experiment

**Figure 3:**
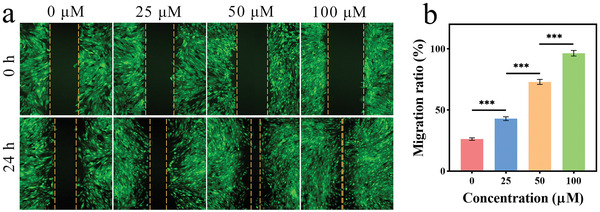
Scratch‐regeneration experiment of FACS. a) Representative fluorescence images of the FACS stained by Calcein‐AM. b) Quantitative analysis image of cell migration ability. Statistical significance was calculated by a two‐tailed Student's *t*‐test, ^***^ *p* < 0.001 (Mean ± SD, *n* = 4).

As mentioned above, it is known that fibronectin plays an important role in the process of wound healing, promoting cell adhesion, migration, proliferation, and hemostasis.^[^ ^15^ ^]^ The scratch‐regeneration experiment was conducted by creating a clear line in the central area of cell growth, after which marginal cells migrate into this area to repair the scratch. The repair rate reflects the rates of cell migration and proliferation, which also indirectly reflects the wound healing capability. Here, scratch‐regeneration experiments on the FACS with different fibronectin concentrations (0, 25, 50, and 100 µm) were performed to determine the effect of fibronectin on cell migration and proliferation. As shown in **Figure** **3**a, these FACS were first stained with Calcein‐AM, and then the scratches (100 µm wide) were created, as indicated by the dotted lines. When these FACS were cultured for an additional 24 h, the scratch spacing gradually narrowed due to cell proliferation and migration. With increasing fibronectin concentration, the spacing decreased more rapidly. The cells' migration capacity could be quantitatively judged by measuring the scratch spacing and calculating the difference at various time points (Figure 3b). Results showed an increase in the cell migration ratio with higher fibronectin concentrations. For example, FACS‐100 µm had the highest migration ratio (96.4%), which was 3.6 times the ratio of the ordinary cell sheet (26.3%), while FACS‐25 µm (42.9%) was only 1.6 times the ratio. Thus, these results confirmed the beneficial effect of fibronectin in enhancing cell migration and proliferation in FACS and also signified its superior capability to promote wound repair.

### 2.6 In Vivo Wound Healing and Mechanism

**Figure 4:**
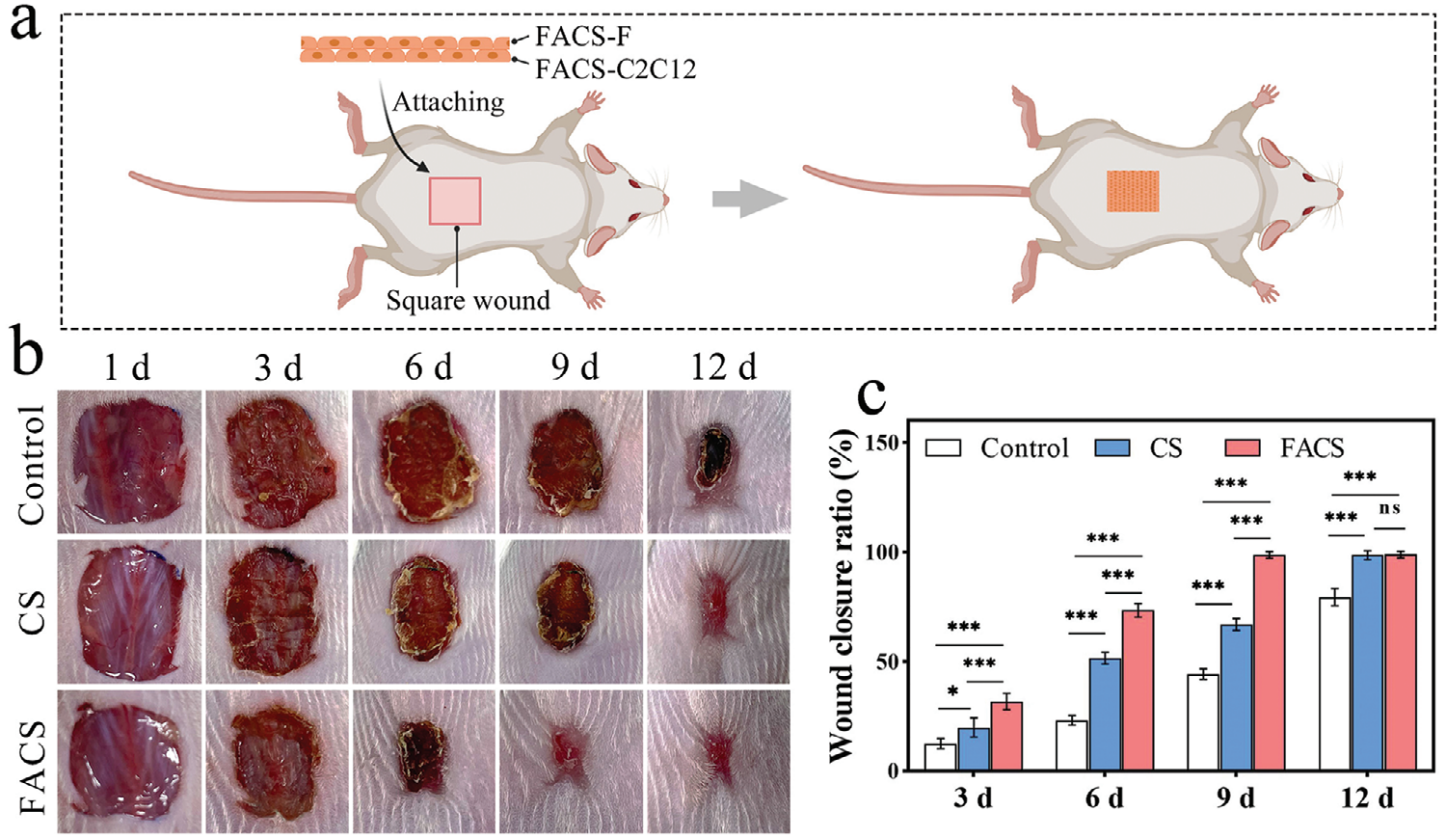
In vivo wound healing experiment. a) Scheme of double cell sheet therapy for wound repair. b) Representative optical images at different time points. c) Wound closure rate. Statistical significance was calculated by a two‐tailed Student's *t*‐test, ^*^ *p* < 0.05, ^***^ *p* < 0.001, ns indicates no significant difference (Mean ± SD, *n* = 4).

To verify the wound repair capability of the FACS, we employed a double cell sheet therapy approach to conduct skin regeneration experiments in animal models. In detail, a square wound measuring 1 × 1 cm was created on the back of the mouse. FACS‐C2C12, composed of the myoblast C2C12 cell sheet, and FACS‐F, composed of the fibroblast cell sheet, were then sequentially applied to the wound (**Figure** **4**a). Concurrently, the therapy was compared to that with unmodified double cell sheets (CS). The effectiveness of the FACS and CS in wound repair was assessed by observing healing progress and measuring closure rates on days 1, 3, 6, 9, and 12 post‐transplantation (Figure 4b,c). The wounds in the CS group had essentially healed by the 12th day, while the closure rate of the Control group without any therapy was only 79.3%, indicating that the cell sheet had a positive effect on promoting wound healing. Moreover, the closure rate of the FACS group reached 31.7% and 73.3% on the 3rd day and 6th day, respectively, and was basically healed on the 9th day, demonstrating a significantly higher closure rate than both the Control and CS groups. These results clearly demonstrated that the FACS had superior wound repair capability, which improved the closure rate to 2.2 times that of the Control group and 1.4 times that of the CS group by the 9th day, indicating the great potential of drug‐assisted cell sheets in tissue repair.

**Figure 5:**
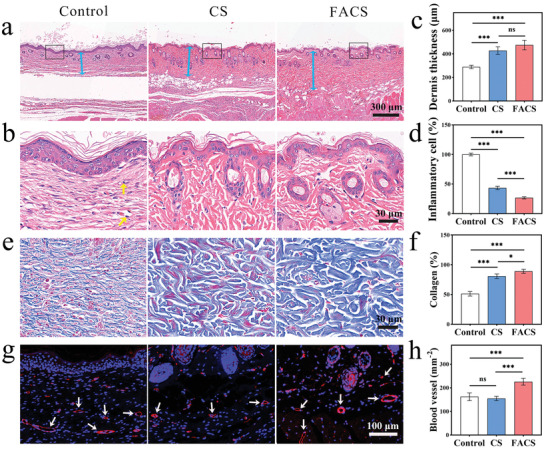
Histological analysis of wound regenerated tissue on the 12th day. H&E staining images of (a) low and (b) high magnification (*n* = 4, blue arrow: dermis, yellow arrow: inflammatory cells). c) The thickness of the dermis. d) The density of inflammatory cells. e) Images for Masson staining and (f) collagen content (100% in normal skin tissue). g) Images for CD31 immunofluorescence staining and (h) blood vessel density (white arrow: blood vessel). Statistical significance was calculated by a two‐tailed Student's *t*‐test, ^*^p < 0.05, ^***^ *p* < 0.001, ns indicates no significant difference (Mean ± SD, *n* = 4).

In the process of skin wound healing, granulation tissue gradually grows and thickens, accompanied by collagen deposition, and inflammatory cell infiltration, and finally regenerates normal skin characteristics, including the epidermis, dermis, and hair follicles.^[^ ^26^ ^]^ On the 12th day, the skin wounds were histologically analyzed using hematoxylin and eosin (H&E) staining, and four sites per section were selected randomly to measure dermis thickness, inflammatory cell density, and regenerated hair follicle density. As illustrated in **Figure** **5**a,b, the complete dermis had formed in both the CS and FACS groups, indicating that the skin tissue in these groups had fully healed, consistent with the previous results. However, the Control group was still transitioning to dermal regeneration, with slender granulation tissue observable at this stage. Statistical analysis revealed that dermis thickness was only 288.9 µm in the Control group, significantly <428.4 µm for the CS group and 475.0 µm for the FACS group (Figure 5c). This result demonstrated that the cell sheet was beneficial to wound healing, especially the FACS. Moreover, the presence of inflammation can impede or even exacerbate wound healing, as indicated by inflammatory cell density. Inflammatory cell infiltration was observed in the Control group, whereas densities in the CS group and FACS group were 43.4% and 26.7% of that in the Control group, respectively, fully reflecting the positive role of the FACS in the anti‐inflammatory response (Figure 5d).

During the formation of the epidermis and dermis, a large number of hair follicles are generated to maintain normal skin secretory functions. Figure S5 (Supporting Information) calculated the density of hair follicles in the regenerated tissues of each group. Among them, only a small number of hair follicles grew at the edge of the wound in the Control group (≈4.7 mm^−2^). By comparation, the follicle density in the CS group and the FACS group increased by 2.6 and 3.6 times, respectively, compared to the Control group, reaching 12.3 mm^−2^ in the CS group and 16.7 mm^−2^ in the FACS group. The results indicated that both the CS and FACS contributed to the regeneration of hair follicles, especially the FACS.

Collagen is the most abundant protein in skin tissue, which promotes the growth of fibroblasts and provides the necessary mechanical properties during tissue regeneration, so it can be used to evaluate wound healing.^[^ ^27^ ^]^ The wound‐regenerated tissues on the 12th day were also analyzed by Masson (MT) staining to assess the wound repair abilities of the FACS. As shown in Figure 5e,f, the large blue areas represented the deposition of collagen. In the Control group, collagen showed a thin fibrous structure, while in the CS group and FACS group, it showed a thick fiber bundle structure, similar to normal skin tissue (Figure S6a, Supporting Information). Based on statistical analysis, it was found that the collagen content in the Control group was only 51.3% of that in normal skin tissue, far lower than the 80.7% in the CS group and 89.0% in the FACS group. These results once again confirmed that the Control group was still in its healing stage on the 12th day, while the CS group and FACS group had already healed due to the contribution of CS and FACS to collagen deposition, especially the FACS.

Moreover, the degree of neovascularization in regenerating tissue is a key indicator of healing. Neovascularization provides sufficient nutrients and oxygen, thus promoting cell proliferation and growth.^[^ ^28^ ^]^ The expression of endothelial connexin was assessed using CD31 immunofluorescence staining to evaluate the neovascularization (Figure 5g,h). The vascular density in the Control group was about 162 mm^−2^, while in the CS group, it was only 154 mm^−2^. This stemmed from the fact that the vascular density of the regenerated tissue decreased gradually with the completion of healing, and the Control group was still in its healing stage. However, the vascular density of the FACS group reached ≈225 mm^−2^ after healing, which was significantly higher than that of both the Control group and the CS group. Notably, the vascular density of normal skin tissue was ≈138 mm^−2^ (Figure S6b, Supporting Information), indicating that the FACS group accelerated nutrient delivery and wound healing by increasing neovascularization density. Moreover, the vessels in the Control group were primarily located around the epidermis, while the CS group and the FACS group had migrated downward to the subcutaneous tissue layer, similar to normal skin. This fully proved that the FACS could stimulate the growth and migration of endothelial cells, thereby accelerating vascular regeneration and wound healing.

To sum up, biocompatible FACS, which combines the advantages of fibronectin and cell sheet in term of wound healing, could enable the regulation of the wound microenvironment, reduce the occurrence of inflammation, accelerate the collagen deposition and wound healing, promote the formation of dermis, hair follicles and vascular regeneration, resulting in improved wound repair effect.

## 3 Discussion

In this work, we prepared fibronectin connecting cell sheets based on click chemistry for wound repair, which represents a pivotal advancement in the realm of wound healing materials. Current research indicates that cell sheets, by emulating the structure and function of native tissue, offer intrinsic advantages in terms of biocompatibility and promoting cell migration and proliferation, which are essential for wound healing.^[^ ^29^ ^]^ However, cell sheets often lack specific bioactive cues that can accelerate the wound‐healing process. By introducing fibronectin through biocompatible click chemistry, we have innovatively combined the benefits of the bioactive molecule fibronectin while circumventing potential cytotoxicity and immunogenicity issues caused by traditional dressings.^[^ ^15^, ^30^ ^]^

As a component of the extracellular matrix, fibronectin not only promotes cell adhesion and tissue regeneration, but also plays a crucial role in the organization of the actin cytoskeleton, extracellular signal transduction, and immune modulation during the repair of damaged tissues.^[^ ^31^, ^32^ ^]^ By incorporating fibronectin into wound dressings, it is possible to simulate the normal extracellular environment, providing a suitable substrate for cell attachment and growth, which is a highly attractive feature for its clinical applications.^[^ ^33^ ^]^ Furthermore, fibronectin has the ability to bind with growth factors and other cytokines, facilitating the local release and activity of these therapeutic agents and thereby enhancing the overall outcome of wound healing.^[^ ^34^ ^]^ This work took advantage of these benefits of fibronectin and efficiently immobilized it onto cell sheets using biocompatible click chemistry, which not only enhanced the stability and bioactivity of fibronectin but also optimized its interaction with cells, laying a solid foundation for more effective wound healing.

Additionally, click chemistry has emerged as a robust platform in bioengineering due to its ability to facilitate precise covalent modifications under mild conditions without compromising biological systems' integrity, which is particularly influential in the design of biofunctional materials.^[^ ^35^ ^]^ In the context of wound healing, the functionalization of biomaterials with signaling molecules like fibronectin is crucial as it mimics the body's natural healing processes, thereby enhancing the efficacy of these materials.^[^ ^36^, ^37^ ^]^ Our adoption of click chemistry to conjugate fibronectin to cell sheets without altering its bioactivity showcases the technique's power and potential for innovative wound‐healing solutions. Another significant advantage of this work is that it enables the customization of cell sheets. The high selectivity and efficiency of click chemistry allow for the introduction of various functional groups onto cell sheets to achieve specific therapeutic functions, such as hemostatic and antimicrobial properties. This strategy not only offers more targeted solutions for treating a variety of complex wounds but also provides robust technical support for designing more sophisticated and multifunctional cell sheet dressings in the future.^[^ ^38^ ^]^

In summary, this approach of modifying fibronectin onto cell sheets using biocompatible click chemistry demonstrates significant novelty and potential impact in the field of wound healing. The enhancement of the wound healing abilities of cell sheets and the introduction of functionalized cell sheets overcome the limitations of conventional wound dressings and hydrogels. The integration of click chemistry offers a versatile and controlled method for modifying the surfaces of cell sheets, thereby expanding the possibilities for customized and targeted therapeutic applications.

## 4 Conclusion

In this study, we harvested cell sheets using NIR‐responsive substrates and bound fibronectin onto their surfaces to prepare FACS through metabolic glycoengineering of unnatural sugars, carbon diimide chemistry and click chemistry. The biological evaluation showed that the binding of fibronectin and the entire biochemical reaction process were biocompatible and further promoted cell migration and proliferation. In vivo wound healing experiments also confirmed that FACS combined the dual therapeutic effects of fibronectin and cell sheets, resulting in superior wound repair capability compared to conventional cell sheets (CS). Specifically, FACS could enhance the regeneration of the dermis/epidermis, hair follicles, and blood vessels, accelerate collagen deposition, reduce inflammatory reactions, and so on, thus demonstrating significant practical potential in regenerative medicine.

## 5 Experimental Section

### Materials

N_3_‐glucose was purchased from Shanghai Yuanye Biotechnology Co. Ltd. DBCO‐PEG_4_‐NHS was acquired from Xi'an Confluore Biotechnology Co. Ltd. AF488 DBCO, 5‐isomer was obtained from Lumiprobe Corporation. Fibronectin was purchased from Yeasen Biotechnology Co. Ltd. Cell counting kit‐8 was obtained from Beyotime Biotechnology Co. Ltd. Single‐walled nanotubes (CNTs) were acquired from Chengdu Organic Chemicals Co. Ltd. Rat tail tendon collagen solution (Type I, 3 mg mL^−1^) was obtained from Dalian Meilun Biotechnology Co. Ltd. The fibroblasts (NIH 3T3) and myoblast cells (C2C12) were kindly provided by the Stem Cell Bank, Chinese Academy of Sciences.

### Preparation of the NIR‐Driven Dynamic Wrinkling Biointerface (Wrinkle‐Col)

The Wrinkle‐Col was prepared according to previously reported work.^[^ ^21^ ^]^ Briefly, the PDMS prepolymer and CNTs were mixed in a 1000:1 ratio for 24 h. After adding the curing agent (10:1 ratio of prepolymer to curing agent) and curing at 70 °C for 4 h, the photothermal responsive PDMS/CNTs substrate was obtained. Additionally, poly (styrene‐co‐perfluorooctyl acrylate) (PSF) was synthesized by free radical copolymerization of styrene and perfluoroalkyl ethyl acrylate at a 4:1 ratio. This reaction was carried out at 70 °C for 12 h. Subsequently, the toluene solution of PSF (3 wt.%) was spin‐coated onto the PDMS/CNTs substrate. After heating to 90 °C for 5 min and cooling to room temperature, the NIR‐responsive dynamic wrinkles were formed. Finally, the Wrinkle‐Col was obtained by coating the thermosoluble Type I collagen (3 mg mL^−1^) onto the surface of the PDMS/CNTs substrate.

### N_3_ Tagging and Characterization of Cell Surfaces

To tag N_3_ on the cell surfaces, 1 mg of N_3_‐glucose was added to 1 mL of medium (DMEM medium for C2C12, MEM medium for Fibroblasts), and the stock solution with a concentration of 2.33 mm was obtained after complete dissolution and mixing. Moreover, fibroblasts were seeded on the Wrinkle‐Col placed in a 24‐well plate at a density of 5 × 10^5^ cells per cm^2^ and cultured at 37 °C under an atmosphere of 5% CO_2_. When the cell density on the Wrinkle‐Col reached 80%, the medium was refreshed in each well. Then, N_3_‐glucose stock solution of 0, 11, 22, and 44 µL were added to prepare N_3_‐glucose solution with concentration gradients of 0, 25, 50, and 100 µm, respectively. These systems were incubated in an incubator with 37 °C and 5% CO_2_ for 48 h to ensure that N_3_ was fully tagged onto the cell surfaces through the glucose metabolism.

To verify the successful tagging of N_3_ on the cell surfaces, the cells were stained with DBCO‐modified fluorescent dye (AF488 DBCO, 5‐isomer). First, the Wrinkle‐Col substrate with cultured cells was rinsed carefully with PBS for 2 times, and then 4% paraformaldehyde solution was added to fix the cells for 10 minutes at room temperature. After removal of the paraformaldehyde, each well was incubated with 1 mL of AF488 DBCO staining solution (100 µm) for 30 min. Subsequently, the staining solution was removed and rinsed 3 times with PBS, and Hoechst33342 was then added for staining for 5 min. Following the same rinsing steps, the fluorescent images were observed using a confocal laser microscope (TCS SP8 STED 3X).

To quantitatively analyze the tagging of N_3_ on the cell surfaces, cells on the Wrinkle‐Col were digested into cell suspension with trypsin. After centrifugation, 1 mL of AF488 DBCO staining solution (100 µm) was added and incubated for 30 min. Then, the staining solution was removed by centrifugation and rinsed with PBS. This step was repeated three times. Finally, the cells were analyzed by flow cytometry.

To evaluate the effect of N_3_ tagging on cell survival rate, a CCK‐8 kit was used. Following the method mentioned above, the N_3_‐glucose stock solution was added to the medium and then incubated with cells for 24, 48, and 72 h, respectively. Subsequently, the fresh medium was replaced and 100 µL of CCK‐8 solution was added to each well. After 1 h of incubation, 100 µL of the supernatant medium was aspirated per well and transferred to a 96‐well plate. The absorbance at different time points of N_3_ incubation was measured with a microplate reader at 490 nm, and the cell survival rate was calculated using the following Equation (1):

(1) $$\mathrm{The}\;\mathrm{cell}\;\mathrm{survival}\;\mathrm{rate}\;\;\left(\%\right)=\frac{I_{n}-I_{b}}{I_{0}-I_{b}}\;\;\times 100\%$$

Where *I*_n_ is the absorbance of N_3_‐glucose incubation for the *n*th hour, *I*_b_ and *I*_0_ are the absorbance of a 96‐well plate and normal cells, respectively.

### The Connection of DBCO and Fibronectin

To modify the fibronectin with DBCO, the derivative DBCO‐PEG_4_‐NHS was selected. Specifically, 100.5 µL of DMSO solution containing DBCO‐PEG_4_‐NHS (3 mm) was added to 2 mL of fibronectin solution (7.5 µm) under continuous stirring and the mixture was allowed to react at room temperature for 24 h. The reaction product was then centrifuged through a centrifugal filter (5000 r/min, 5 min), rinsed with sterile ultrapure water, and the above operation was repeated 3 times to obtain purified DBCO‐Fibronectin. Finally, the DBCO‐Fibronectin was lyophilized to remove any remaining water.

### The Attachment of the Fibronectin to Cell Surfaces

After cells on the Wrinkle‐Col substrate in a 24‐well plate were tagged with gradient concentrations of N_3_, 1 mL of fresh medium was added to each well. To accurately control and optimize the attachment of fibronectin, the additional concentration of DBCO‐Fibronectin was consistent with that of the N_3_ tagging. Consequently, 5.25, 10.5, and 21.0 mg of DBCO‐Fibronectin were added to the corresponding wells, resulting in final concentrations of 25, 50, and 100 µm, respectively, after thorough mixing. After incubation at room temperature for 30 min, the mixture was suctioned and rinsed with PBS. The above steps were repeated 3 times to remove any unattached DBCO‐Fibronectin. Following the same protocol, a CCK‐8 kit was used again to evaluate the effects of the DBCO‐Fibronectin concentration and incubation time on the cell survival rate.

### The Harvesting and Characterization of FACS

When the fibronectin‐attached cells on the Wrinkle‐Col substrate reached 90% density, FACS could be harvested using a photothermal response strategy previously reported.^[^ ^21^ ^]^ Specifically, the Wrinkle‐Col substrate with cells was exposed to 20s‐on/5s‐off cycles of NIR irradiation (808 nm, 3.5 W cm^−2^), and the FACS would gradually peel off from the Wrinkle‐Col surface within 10 min.

Furthermore, the viability of FACS after harvesting was assessed using acridine orange (AO, live cells) and ethidium bromide (EB, dead cells) double staining. Specifically, the FACS was transferred to a new culture dish and incubated with AO/EB mixed solution (5 µg mL^−1^, 1 mL) for 5 min. After careful rinsing with PBS, the fluorescent images were observed using an inverted fluorescence microscope (TS‐2000, Nikon).

To assess the states of intercellular connection and nucleus, the FACS was initially fixed with 4% paraformaldehyde solution for 10 minutes. Then, the FACS was rinsed with 0.1% Triton X‐100 for 10 min and incubated with 5% BSA as a blocking solution for 30 min. Subsequently, it was stained with 1 mL of anti‐N‐cadherin solution (5 µg mL^−1^) at room temperature in the dark for 45 min. After careful rinsing 3 times with 0.1% Triton X‐100, 1 mL of DAPI solution (5 µg mL^−1^) was added and incubated with FACS for 10 min. After removal of the DAPI solution and rinsing with PBS 3 times, the fluorescent images were collected via an inverted fluorescence microscope (TS‐2000, Nikon).

To characterize the attaching of fibronectin, the FACS was examined using SEM. Specifically, the FACS was initially rinsed with PBS 3 times and then fixed with 4% paraformaldehyde solution for 20 min. Following the same rinsing protocol, the FACS was dehydrated sequentially with 30%, 50%, 75%, 80%, 95%, and 100% ethanol solutions for 15 min each time. The dehydrated FACS was immersed in ethyl acetate for 30 min. Subsequently, it was dried using critical point drying. The dried FACS was then sputter‐coated with gold, and SEM imaging was conducted for detailed observation.

### Scratch‐Regeneration Experiment

The harvested fibroblast FACS was first spread out in 6‐well plates for culturing. After cell adhesion occurred, the culture medium was removed and the FACS was stained with Calcin‐AM (2 µg mL^−1^, 1 mL) in the dark for 20 min. Subsequently, the staining solution was removed and gently rinsed with PBS 3 times, and then a scratch ≈100 µm wide was created on the FACS using a pipette tip. The gap closure was monitored using an inverted fluorescence microscope (TS‐2000, Nikon) at 0, 24, and 48 h after wounding. The cell migration ratio was calculated by the following Equation (2):

(2) $$\mathrm{The}\;\mathrm{cell}\;\mathrm{migration}\;\mathrm{ratio}\;\;\left(\%\right)=\frac{L_{0}-L_{t}}{L_{0}}\;\times 100\%$$

Where *L*_0_ is the initial scratch spacing, *L*_t_ is the scratch spacing after incubation for *t* h.

### In Vivo Wound Healing Experiment

The used mice (Balb/c) to evaluate the wound repair capability of the FACS, and it was approved by the animal ethics committee of Shanghai Jiao Tong University (approval number: A2023033). The mice were divided into three groups: control group (Control), unmodified cell sheet group (CS), and fibronectin‐attached cell sheet group (FACS), with 4 mice in each group. Among them, the Control group did not receive any treatment, the CS group was treated with unmodified double cell sheets, and the FACS group was treated with fibronectin‐attached double cell sheets (100 µm). In detail, three groups of mice were anesthetized with 10% chloral hydrate after 5 days of feeding, and then a square‐shaped wound measuring 1 × 1 cm was surgically created on their back. According to the above method, the FACS‐C2C12 composed of myoblast C2C12 cell sheet and the FACS‐F composed of fibroblast cell sheet were simultaneously prepared and applied to the wounds in sequence as treatment. The wound healing process was monitored and photographed, and the wound area was measured using ImageJ software. The wound closure rate was calculated using the following Equation (3):

(3) $$\mathrm{The}\;\mathrm{wound}\;\mathrm{closure}\;\mathrm{rate}\;\;\left(\%\right)=\frac{S_{0}-S_{t}}{S_{0}}\;\times 100\%$$

Where *S*_0_ is the initial wound area, *S*_t_ is the wound area on day *t*.

Upon wound healing, the mice were euthanized by excessive carbon dioxide inhalation followed by cervical dislocation. Tissue samples measuring 1.5 × 1.5 cm were excised from around the healed wound area, fixed in 4% paraformaldehyde, and incubated overnight at 4 °C. Subsequently, these samples were paraffin‐embedded and sectioned (4 µm thick) for histological analysis. The sections were deparaffinized in anhydrous xylene, followed by hydration in gradient concentrations of ethanol solutions (100%, 90%, and 70%). The processed sections were stained with H&E and MT, and the staining results were observed with an ordinary light microscope.

To further observe the expression of endothelial connexin, immunofluorescence staining was performed using an anti‐mouse CD31 antibody. First, the paraffin‐embedded sections were subjected to antigen retrieval treatment, incubated in EDTA antigen retrieval solution (pH 9.0) at room temperature, and then added to 5% BSA to block for 1 h. After completing the above steps, an anti‐mouse CD31 antibody (diluted 300x in 1% BSA) was added and incubated overnight at 4 °C. Then, CY3 (red fluorescence) labeled anti‐rabbit IgG (diluted 300x in 1% BSA) was added as a secondary antibody and incubated for 50 min. After rinsing with PBS 3 times, DAPI staining solution was added and incubated in the dark for 10 min. After rinsing again, the sections were sealed, and the fluorescence images were collected via an inverted fluorescence microscope (TS‐2000, Nikon).

### Statistical Analysis

The data analysis was performed using a GraphPad Prism 8.0 software and presented as mean ± SD. Statistical significance was calculated by a two‐tailed Student's *t*‐test (*n* = 4), and the probability including ^*^ *p* < 0.05, ^**^ *p* < 0.01, ^***^ *p* < 0.001 and ns, respectively, denotes significant, very significant, highly significant and no significant.

## Conflict of Interest

The authors declare no conflict of interest.

## Supporting information

Supporting Information

## Data Availability

The data that support the findings of this study are available in the supplementary material of this article.
