# Supplementary material for: Fibronectin Connecting Cell Sheet Based on Click Chemistry for Wound Repair
Source: Adv Sci (Weinh). 2024 Jan 2;11(11):2306746. doi: 10.1002/advs.202306746 (PMC10953575; doi:10.1002/advs.202306746)
Supplement: Supplementary file 1 — Supporting Information [file ADVS-11-2306746-s001.pdf]

ADVANCED SCIENCE
Open Access

# Supporting Information

for *Adv. Sci.*, DOI 10.1002/advs.202306746

Fibronectin Connecting Cell Sheet Based on Click Chemistry for Wound Repair

*Wei Xu, Meng He and Qinghua Lu**

# Supporting Information

**Fibronectin connecting cell sheet based on click chemistry for wound repair**

*Wei Xu, Meng He and Qinghua Lu**

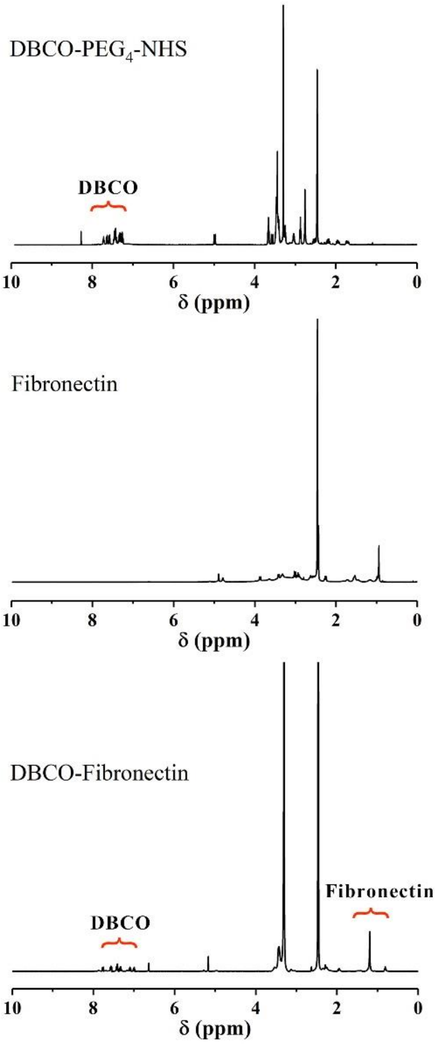


**Figure S1**. The NMR analysis of DBCO-PEG$_4$-NHS, Fibronectin and DBCO-Fibronectin.

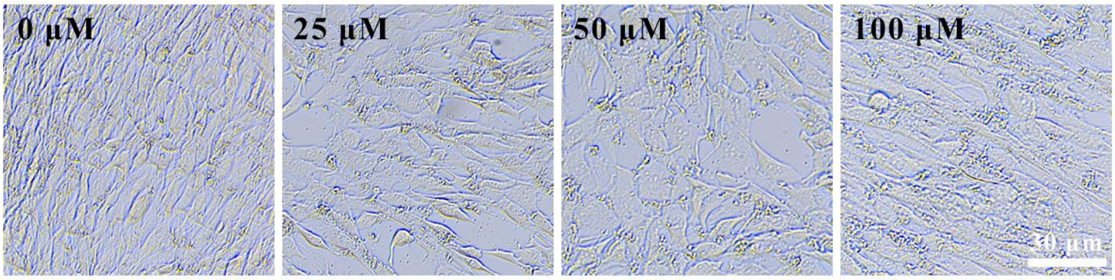


**Figure S2**. Optical images of DBCO-Fibronectin tagged on the cell surfaces with different concentrations.

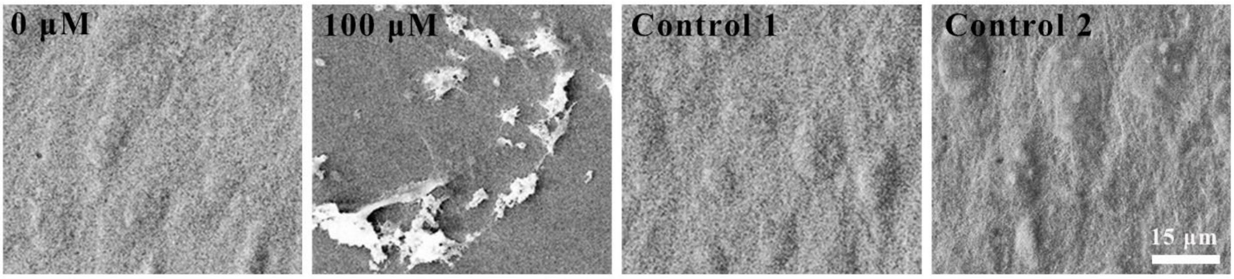


**Figure S3**. SEM images of surface topography: cells tagged with $N_3$ incubated with DBCO-Fibronectin (0 μM and 100 μM) for 30 minutes; cells tagged with $N_3$ incubated with fibronectin that was not modified by DBCO (Control 1, 100 μM) for 30 minutes; cells not tagged with $N_3$ incubated with DBCO-Fibronectin (Control 2, 100 μM) for 30 minutes.

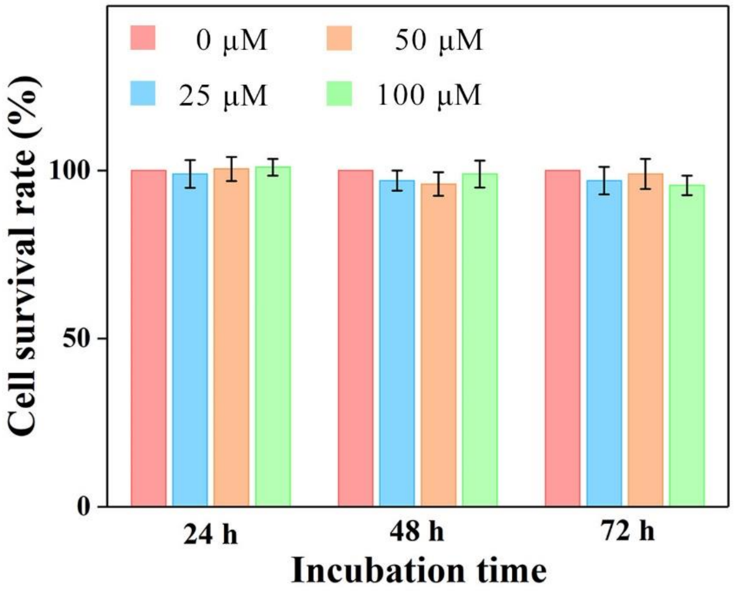


**Figure S4**. Cell survival rate at different DBCO-Fibronectin incubation time points measured via CCK-8.

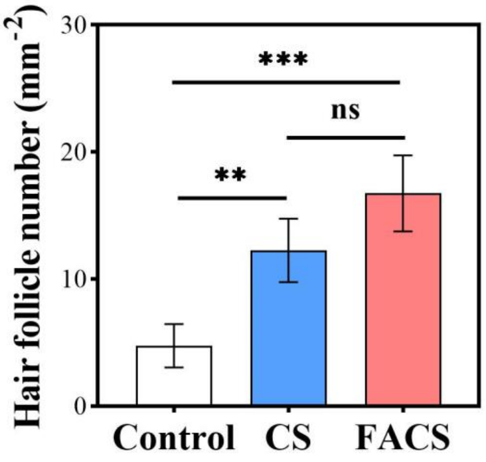


**Figure S5**. The density of regenerated hair follicle of wound regenerated tissue on the 12th day. Statistical significance was calculated by a two-tailed Student's t-test, $^{**}p < 0.01$, $^{***}p < 0.001$, ns indicates no significance difference.

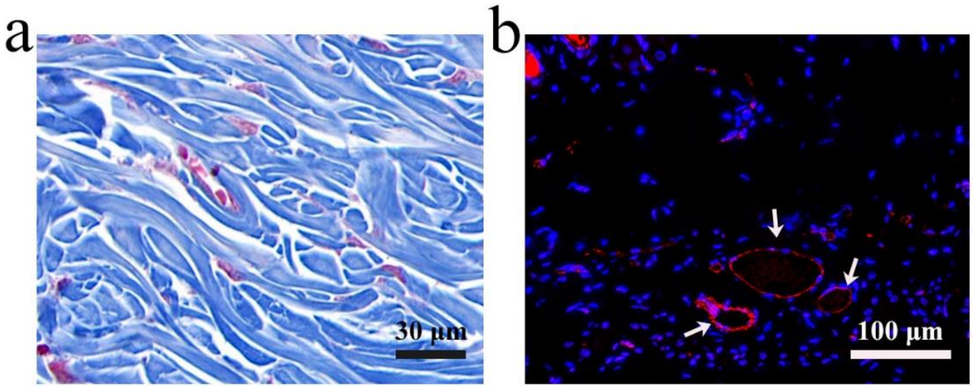


**Figure S6**. (a) Collagen content of the normal skin tissue. (b) Images for CD31 immunofluorescence staining of the normal skin tissue (white arrow: blood vessel).
